# Development of risk prediction models for glioma based on genome-wide association study findings and comprehensive evaluation of predictive performances

**DOI:** 10.18632/oncotarget.10882

**Published:** 2016-07-28

**Authors:** Yingjie Zhao, Gong Chen, Hongjie Yu, Lingna Hu, Yunmeng Bian, Dapeng Yun, Juxiang Chen, Ying Mao, Hongyan Chen, Daru Lu

**Affiliations:** ^1^ State Key Laboratory of Genetic Engineering and MOE Key Laboratory of Contemporary Anthropology, Collaborative Innovation Center for Genetics and Development, Institute of Genetics, School of Life Sciences, Fudan University, Shanghai, China; ^2^ Neurosurgery Department of Huashan Hospital, Fudan University, Shanghai, China; ^3^ Center for Genetic Epidemiology, School of Life Sciences, Fudan University, Shanghai, China; ^4^ Department of Neurosurgery, Changzheng Hospital, Second Military Medical University, Shanghai, China

**Keywords:** glioma, genome wide association study, risk prediction, genetic risk score, prediction risk from logistic regression analyses

## Abstract

Over 14 common single nucleotide polymorphisms (SNP) have been consistently identified from genome-wide association studies (GWAS) as associated with glioma risk in European background. The extent to which and how these genetic variants can improve the prediction of glioma risk has was not been investigated. In this study, we employed three independent case-control datasets in Chinese populations, tested GWAS signals in dataset1, validated association results in dataset2, developed prediction models in dataset2 for the consistently replicated SNPs, refined the consistently replicated SNPs in dataset3 and developed tailored models for Chinese populations. For model construction, we aggregated the contribution of multiple SNPs into genetic risk scores (count GRS and weighed GRS) or predicted risks from logistic regression analyses (PRFLR).

In dataset2, the area under receiver operating characteristic curves (AUC) of the 5 consistently replicated SNPs by PRFLR(SNPs) was 0.615, higher than those of all GRSs(ranging from 0.607 to 0.611, all *P*>0.05). The AUC of genetic profile significantly exceeded that of family history (fmc) alone (AUC=0.535, all *P*<0.001). The best model in our study comprised “PRURA +fmc” (AUC=0.646) in dataset3. Further model assessment analyses provided additional evidence.

This study indicates that genetic markers have potential value for risk prediction of glioma.

## INTRODUCTION

Gioma makes up 80% of all malignant brain tumors in adults [1]. Genetic predisposition to glioma is well known in the settings of rare familial tumor syndromes [2]. To date, five GWAS reports have been published and have led to the discovery of about 14 SNPs in 7 chromosome regions associated with glioma risk for individuals of European descent [3-7]. Encouragingly, these independent GWAS have identified several susceptibility SNPs in common with one another. For example, Rajaraman et al. successfully replicated 8 signals reported in another GWAS in Caucasian populations, with all associations in the same direction as reported in the original study [7]. Our group replicated 5 of these association signals in 20q13.33, 11q23.3 and 5p15.33 within a Chinese population [8]. This consistency across studies highlights the robustness of GWAS design and promises to unlock the underlying genetic architecture of glioma by identifying loci that may play a role in the etiology of glioma. Although each of the variants is only moderately associated with glioma risk (each with an 18% to 60% increase in the relative odds ratio per risk allele), the alleles collectively have a strong dose-dependent effect [8].

The success of GWAS has greatly facilitated risk prediction by providing ever-increasing disease risk-associated single nucleotide polymorphism (SNPs), most of which were well validated and replicated by independent studies [9, 10]. These advances comprise a vital step toward realizing the goals of personalized medicine. Several of the resultant genetic prediction models have been developed, validated and evaluated across a large spectrum of diseases [11-20]. However, in the context of glioma, it remains unclear as to whether the combination of SNP genotypes and family history provide added benefit in risk prediction. To address these issues, we have employed three relatively large case-control datasets, genotyping all glioma risk-associated SNPs identified from GWAS in dataset1 and 2 and testing their associations with glioma risk. Then for those consistently replicated SNPs (associations in the same direction and both *P* values < 0.05), we assessed predictive performance using three different methods for estimating the combined value of genetic variants. For more specific tailored prediction in Chinese population, we genotyped SNPs in larger regions surrounding the consistently replicated SNPs in a larger region and evaluated prediction performance of the combination of all independent risk-associated loci across the susceptible regions in dataset3. Finally, we examined the calibration and discrimination features of the genetic models using Hosmer-lemeshow “goodness-of-fit” tests (H-L test) and AUCs. To gain further insight into the value added by incorporation of genetic information into risk prediction models, we employed continuous Net reclassification improvement (cNRI) and Integrated discrimination improvement (IDI) analyses.

## RESULTS

Characteristics of the subjects within each of the three datasets, along with histologic subtypes of cases are shown in Supplementary Table 1. Cases and controls were adequately well-matched in terms of age and sex in dataset2 and 3 (All *P* >0.05), with no significant differences in the distribution of smoking status between cases and controls (All *P* >0.05).

A Positive family history of cancer (fmc) was defined as having a first-degree relative with a pathological diagnosis of cancer. Cases were more likely than controls to report fmc. Among clinical variables, only fmc demonstrated an association with glioma risk in univariate analysis in dataset2 and 3 (OR =1.63, 95%CI=1.24-2.15, *P*= 0.001 and OR=1.47 95%CI=1.13-1.91, *P*= 0.004, respectively for dataset2 and 3) and was therefore used to build a baseline risk model for glioma (Supplementary Table 2). No significant differences were observed between subjects excluded from the study due to data missing and those included. This suggests that bias has not been introduced into the following data analysis as a result of exclusion of missing data (data not shown).

**Figure 1:**
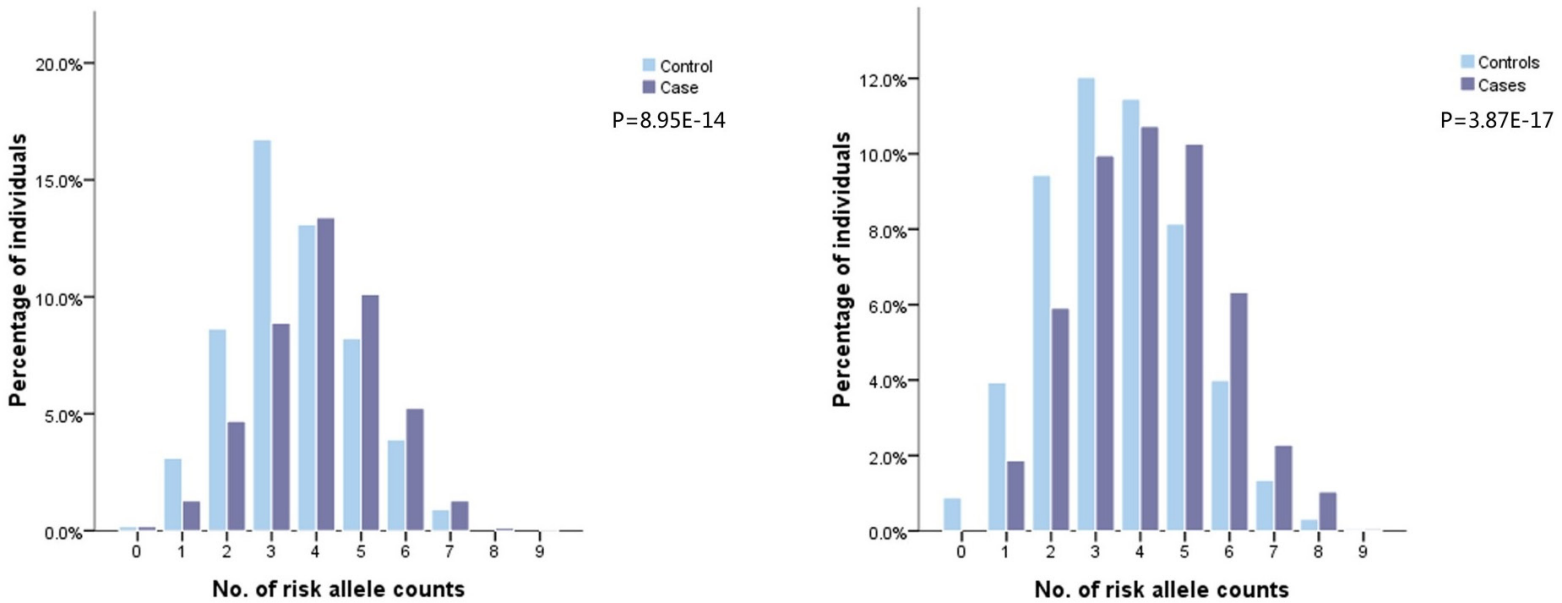
Frequency distribution of number of risk alleles in glioma cases and controls in dataset2 and 3 Footnotes: Five SNPs included in dataset 2: rs2736100 at 5p15.33, rs2157719 at 9p21.3, rs498872 at 11q23.3, rs6010620 at 20q13.33, rs1077236 at 8q24.21. Seven SNPs included in dataset3:rs2853677 and rs2735948 at 5p15.33, rs6589664, rs494560 and rs17748 at11q23.3, rs3761121 and rs1058319 at 20q13.33.

Detailed information about selected SNPs and their associations with glioma risk across dataset1 and 2 are presented in Supplementary Table 3. Two SNPs in EGFR were not available in dataset2. Eight SNPs were significantly associated with glioma risk in dataset1 (*P*<0.05). Of these eight SNPs, six were consistently replicated in dataset2 (rs2736100 at 5p15.33; rs2157719 and rs1412829 at 9p21.3; rs498872 at 11q23.3; rs6010620 and rs4809324 at 20q13.33), all of which were common in Chinese population (risk allele frequencies 0.111-0.688). Among these, rs1412829 was removed from subsequent analyses for two reasons. First, it is in complete linkage disequilibrium (LD) with rs2157719 (pairwise r^2^ = 1 in Chinese population). Second, association strength of rs1412829 with glioma risk was more significant than that of rs2157719 (9.23E-03 vs. 0.025). Rs4809324 was also removed because it failed to survive in a multivariate logistic regression analysis, due to dependency with rs6010620 (pairwise r^2^ = 0.334 in Chinese population). The remaining four SNPs were used for model construction in dataset2. Rs1077236 at 8q24.21 was also included in the model given its potential for robust association with glioma risk in Chinese populations (Table 1). Detailed information of the 42 SNPs selected in dataset3, and their associations with glioma risk from univariate regression analysis are shown in Supplementary Table 4. Twenty of these were significantly associated with glioma risk, but ten were removed from further analyses due to LD (pairwise r^2^ ≥ 0.35) and less significances (relatively lager *P* values). Ten selected SNPs were then pooled in a multivariate logistic regression analysis using a backward likelihood ratio method [21, 22]. Three were filtered out and seven (rs2853677 and rs2735948 at 5p15.33; rs6589664, rs494560 and rs17748 at11q23.3; rs3761121 and rs1058319 at 20q13.33) retained for model construction in dataset3, all of which were common (risk allele frequencies 0.146-0.746, Table 2). The number of risk allele counts was normally distributed among cases and controls and was skewed to the right for cases in both datasets. Higher mean counts were seen in the cases group (4.05±1.40 vs. 3.54±1.37, *P*= 8.95E-14 in dataset2; 4.14±1.60 vs. 3.52±1.59, *P* = 3.87E-17 in dataset3, respectively, Figure 1A and 1B).

**Table 1:** Five consistently replicated SNPs for model development in dataset2

SNP	CHR.	Nearest gene	Region	Location on Chromosome^a^	Non-risk	Risk	Dataset1					Dataset2			
							Risk allele frequency			OR (95%CI)^b^	*P* value^b^	Risk allele frequency		OR (95% CI)^b^	*P* value^b^
							Cases	Controls				Cases	Controls		
rs2736100	5	*TERT*	Intron	1339516	T	G	0.479		0.413	1.30(1.17-1.46)	3.96E-06	0.482	0.418	1.29(1.13-1.49)	2.69E-04
rs1077236	8	*CCDC26*	Intergenic	130709683	A	C	0.698		0.677	1.10(0.95-1.28)	0.219	0.725	0.688	1.20(1.03-1.39)	0.021
rs2157719	9	*CCDKN2A/B*	Intron	22023366	T	C	0.140		0.113	1.28(1.08-1.51)	4.19E-03	0.141	0.111	1.32(1.07-1.62)	9.23E-03
rs498872	11	*PHLDB1*	UTR-5	117982577	A	G	0.303		0.272	1.23(1.08-1.39)	1.19E-03	0.349	0.285	1.35(1.16-1.56)	7.81E-05
rs6010620	20	*RTEL1*	Intron	61780283	T	C	0.304		0.266	1.21(1.07-1.37)	2.39E-03	0.330	0.267	1.35(1.16-1.57)	9.25E-05

a, based on NCBI Build 36; b, Odds ratios (ORs), 95% confidence interval (95%CI) and P values were calculated from univariate logistic regression analyses based on additive model.

**Table 2:** Seven independent SNPs for model development in dataset3

SNP	CHR	Nearest gene	Region	Location on Chromosome^a^	Non-risk	Risk	Risk allele frequency		OR (95%CI)^b^	*P* value^b^
							Cases	Controls		
rs2853677	5	*TERT*	Intron	1287194	T	C	0.449	0.375	1.36(1.20-1.55)	2.70E-06
rs2735948	5	*TERT*	Intergenic	1299213	C	T	0.170	0.146	1.20(1.01-1.42)	0.044
rs6589664	11	*TMEM25*	Exon	117910014	G	A	0.310	0.271	1.21(1.05-1.39)	6.80E-03
rs494560	11	*PHLDB1*	*Intron*	118026759	A	G	0.801	0.746	0.73(1.18-1.60)	4.04E-05
rs17748	11	*PHLDB1*	*UTR-3*	118033634	C	T	0.327	0.263	1.36(1.18-1.56)	1.57E-05
rs3761121	20	*ZGPAT*	Intron	62342695	A	G	0.269	0.202	1.45(1.25-1.69)	9.85E-07
rs1058319	20	*SLC2A4RG*	UTR-3	62374389	T	C	0.354	0.256	1.59(1.39-1.83)	4.76E-11

a, based on NCBI Build 36; b, ORs, 95%CI and *P* values were calculated from univariate logistic regression analysis based on additive model.

Associations between glioma risk and genetic risk score derived from three different methods, cGRS, wGRS1 and wGRS2 (count Genetic Risk Score, weight Genetic Risk Score1 and weight Genetic Risk Score2, respectively) are shown in Table 3 for dataset2. For cGRS, we first classified subjects into six groups based on the number of risk alleles they harbored (≤1, 2, 3, 4, 5, and ≥6 number of risk alleles) and calculated the corresponding odds ratios (OR) and 95% confidence intervals (CI) relative to the first group. As is shown in Table 3, glioma risk increases with increasing of risk allele counts (*P* for trend=2.73E-12). Subjects carrying ≥6 of the risk alleles (14.8% of cases and 8.78% of controls) had a 2.13-fold (95%CI=1.79-5.49, *P*=6.62E-05) increased risk of developing glioma compared with those carrying ≤1 of the risk alleles (6.00% of cases and 3.03% of controls). For evaluating the risk of wGRS1 and wGRS2, we classified subjects into four equally-sized groups by quartiles determined from controls. Compared with individuals who were in the lowest quartile, those in the highest quartile had a 1.77-fold (95%CI=2.10-3.65, *P*=7.67E-13) increased risk of glioma for wGRS1 and 1.78-fold (95%CI=2.10-2.65, *P*= 6.25E-13) increased risk for wGRS2. Similar and consistent results were observed for the seven independent SNPs in dataset3 (Table 4). Notably, subjects carrying ≥7 risk alleles (6.96% of cases and 3.32% of controls) had a 4.09-fold (95%CI=2.88-8.99, *P*=2.07E-08) increased risk for developing glioma compared with those carrying ≤1 risk alleles (3.85% of cases and 9.85% of controls). We estimated interaction between each SNP pair in dataset2 and 3. Except for one probably spurious association (*P* = 0.031 for rs2735948 and rs376112 in dataset3, false discovery rate adjusted *P* = 0.413), the results demonstrated no evidence of interaction between any of the SNP pairs for both datasets (Supplementary Tables 5 and 6).

**Table 3:** Association between the cumulative effect of 5 independent SNPs and glioma risk in dataset2

Risk prediction models	Cases (%)	Controls (%)	OR(95%CI)	*P* value	Trend *P* value
	743	900			
counts Genetic Risk Score (cGRS)					
0-1	24(3.23)	54(6.00)	1.00(reference)		
2	77(10.36)	142(15.78)	1.22(0.70-2.13)	0.482	
3	146(19.65)	275(30.56)	1.20(0.71-2.01)	0.504	
4	220(29.61)	215(23.89)	2.30(1.37-3.86)	0.002	
5	166(22.34)	135(15.00)	2.77(1.63-4.71)	1.76E-04	
≥6	110(14.80)	79(8.78)	3.13(1.79-5.49)	6.62E-05	2.73E-12
weighed Genetic Risk Score (wGRS)1					
0(<Q25)	120(16.15)	259( 28.78)	1.00(reference)		
1(Q25∼Q50)	107(14.40)	198(22.00)	1.17(0.85-1.61)	0.345	
2(Q50∼Q75)	229(30.82)	219(24.33)	2.26(1.70-3.00)	2.14E-08	
3(≥Q75)	287(38.63)	224(24.89)	2.77(2.10-3.65)	7.67E-13	3.63E-15
weighed Genetic Risk Score (wGRS)2					
0(<Q25)	120(16.15)	259(28.78)	1.00(reference)		
1(Q25∼Q50)	107(14.40)	199(22.11)	1.16(0.84-1.60)	0.361	
2(Q50∼Q75)	226(30.42)	219(24.33)	2.24(1.68-2.98)	3.12E-08	
3(≥Q75)	287(39.03)	223(24.78)	2.78(2.10-3.70)	6.25E-13	3.13E-15

**Table 4:** Association between the cumulative effect of the 7 independent SNPs and glioma risk in dataset3

Risk prediction models	Cases (%)	Controls (%)	OR(95%CI)	*P* value	Trend *P* value
	934	995			
counts Genetic Risk Score (cGRS)					
0-1	36 (3.85)	93(9.35)	1.00(reference)		
2	114 (12.21)	182(18.29)	1.62(1.03-2.54)	0.036	
3	192 (20.56)	232(23.32)	2.14(1.39-3.29)	0.001	
4	207 (22.16)	221(22.21)	2.42(1.58-3.72)	5.39E-05	
5	198(21.20)	157(15.78)	3.26(2.10-5.05)	1.26E-07	
6	122(13.06)	77(7.74)	4.09(2.54-6.61)	8.06E-09	
≥7	65(6.96)	33(3.32)	5.09(2.88-8.99)	2.07E-08	2.58E-12
weighed Genetic Risk Score (wGRS)1					
0(<Q25)	133(14.24)	250(25.13)	1.00(reference)		
1(Q25∼Q50)	191(20.45)	252(25.33)	1.43(1.07-1.89)	0.014	
2(Q50∼Q75)	267(28.59)	257(25.83)	1.95(1.49-2.56)	1.33E-06	
3(≥Q75)	343 (36.72)	235(23.62)	2.74(2.09-3.59)	1.55E-13	2.85E-13
weighed Genetic Risk Score (wGRS)2					
0(<Q25)	130(13.92)	250(25.13)	1.00(reference)		
1(Q25∼Q50)	192 (20.56)	249(25.03)	1.48(1.12-1.97)	0.006	
2(Q50∼Q75)	262(28.05)	251(25.23)	2.00(1.53-2.64)	6.01E-07	
3(≥Q75)	350 (37.47)	244(24.52)	2.76(2.11-3.61)	1.08E-13	3.08E-13

**Figure 2:**
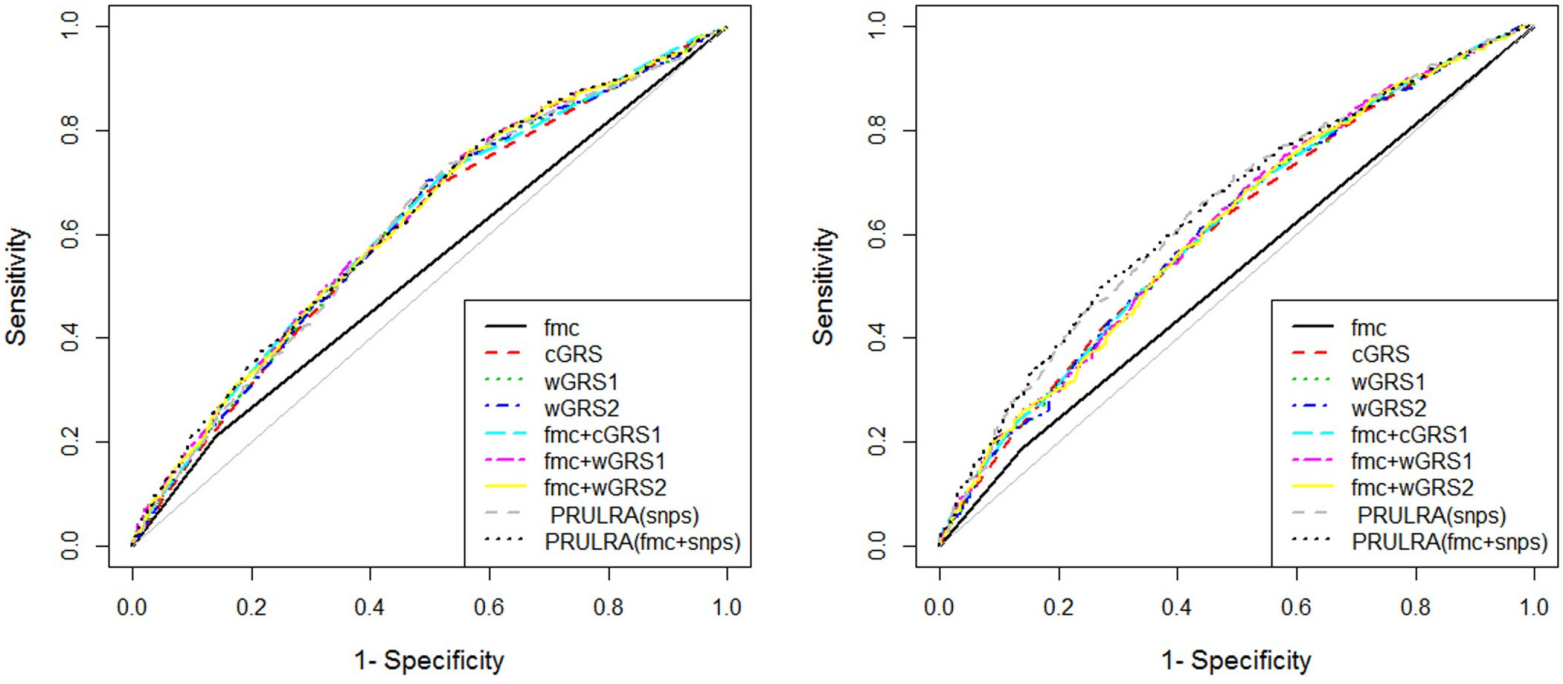
Receiver operating characteristic curve plots in dataset2 and 3 Footnotes: fmc: family history of caner; cGRS: count genetic risk score; wGRS: weighed genetic risk score; PRFLR: predicted risks from logistic regression analysis.

We first assessed the overall performance of risk prediction models using AUC statistics. As shown in Table 5 and Figure 2, the ability of fmc to discriminate cases from controls was 0.535 (95% CI=0.515-0.554). This performance was only slightly better than random classification in dataset2. The AUCs for wGRS1 and wGRS2 were almost identical, with 0.610 for wGRS1 and 0.611 for wGRS2 (*P* = 1.00, Table 6). These were both slightly higher than that of cGRS (AUC = 0.607), though these differences were not statistically significant (both *P* = 0.766). The AUC for PRFLR(SNPs) was 0.615. This was higher than all GRSs, though once again, the differences were not statistically significant (all *P* > 0.05). When we combined fmc and genetic information within one risk model, AUCs increased correspondingly. The higher AUC of the GRS and fmc combination was observed for wGRS1+fmc (0.623), wGRS2+fmc (0.621) and cGRS+fmc (0.620). The highest AUC observed was for PRFLR(SNPs+fmc)(0.625). This, however, was not significantly different compared with AUCs for models incorporating genetic information alone (all *P* > 0.05).

**Table 5:** Prediction performance of genetic risk score and family history for glioma risk

Datasets	No. of subjects	AUC(95%CI)^a^	H-L test^b^ *P* value
**Dataset2**			
fmc	1453	0.535(0.515-0.554)	1.000
cGRS	1643	0.607(0.581-0.644)	0.386
wGRS1	1643	0.610(0.583-0.638)	0.111
wGRS2	1643	0.611(0.584-0.639)	0.049
PRFLR(SNPs)	1453	0.615(0.586-0.644)	0.051
cGRS+fmc	1453	0.620(0.591-0.649)	0.816
wGRS1+fmc	1453	0.623(0.595-0.652)	0.334
wGRS2+fmc	1453	0.621(0.592-0.650)	0.117
PRFLR(SNPs+fmc)	1453	0.625(0.596-0.653)	0.250
**Dataset3**			
fmc	1718	0.526(0.508-0.543)	1.000
cGRS	1921	0.605(0.580-0.629)	0.997
wGRS1	1921	0.607(0.582-0.632)	0.880
wGRS2	1921	0.608(0.583-0.633)	0.113
PRFLR (SNPs)	1718	0.635(0.610-0.660)	0.927
cGRS+fmc	1718	0.611(0.585-0.637)	0.743
wGRS1+fmc	1718	0.611(0.585-0.638)	0.154
wGRS2+fmc	1718	0.609(0.583-0.636)	0.016
PRFLR(SNPs+fmc)	1718	0.646(0.619-0.672)	0.393

AUC: the area under operating characteristic curves; fmc: family history of caner; cGRS: count genetic risk score; wGRS: weighed genetic risk score; PRFLR: predicted risks from logistic regression analysis; a, 2000 bootstrap replicates; b, Hosmer-Lemeshow “goodness-of-fit” test for model calibration.

**Table 6:** Comparisons of AUC pairs in dataset2 and 3

Datasets	cGRS	wGRS1	wGRS2	PRFLR(SNPs)	cGRS+fmc	wGRS1+fmc	wGRS2+fmc	PRFLR(SNPs+fmc)
**Dataset2**								
fmc	5.30E-06	8.59E-06	8.85E-06	8.63E-06	5.34E-09	5.77E-10	3.22E-10	2.44E-10
cGRS		0.766	0.766	0.738	0.421	0.191	0.392	0.150
wGRS1			1.000	0.738	0.421	0.191	0.392	0.150
wGRS2				0.512	0.413	0.190	0.385	0.150
PRFLR(SNPs)					0.423	0.201	0.396	0.141
cGRS+fmc						0.270	0.739	0.212
wGRS1+fmc							0.340	0.507
wGRS2+fmc								0.224
**Dataset3**								
fmc	4.91E-07	3.52E-07	2.78E-07	2.85E-13	3.10E-10	2.73E-10	4.69E-11	2.20E-16
cGRS		0.501	0.391	3.11E-04	0.229	0.309	0.583	9.07E-05
wGRS1			0.484	1.91E-03	0.554	0.381	0.747	4.37E-04
wGRS2				1.90E-03	0.656	0.497	0.84	4.20E-04
PRFLR(SNPs)					1.71E-03	3.52E-03	2.81E-03	0.280
cGRS+fmc						0.863	0.769	2.33E-04
wGRS1+fmc							0.451	6.38E-04
wGRS2+fmc								2.90E-04

Results are denoted by *P* values of differences in AUC pairs; *P* values in each cell denotes comparison of AUC in corresponding row over that of the corresponding column; Bootstrap method proposed by Delong and his colleagues was used to calculated *P* values; AUC: the area under operating characteristic curves; fmc: family history of caner; cGRS: count genetic risk score; wGRS: weighed genetic risk score; PRFLR: predicted risks from logistic regression analysis.

To generate tailored risk models for the Chinese population and possibly validation the findings in dataset2, we also calculated corresponding AUCs and *P* values for pairwise comparisons in dataset3 (Table 5 and Figure 2). The AUC estimates for fmc, cGRS, wGRS1, wGRS2, cGRS+fmc, wGRS1+fmc and wGRS2+fmc were roughly equivalent to those from dataset2, suggesting that these values were stable with no evidence of model overfitting. In contrast, AUCs of PRFLR(SNPs) and PRFLR(SNPs+fmc) were remarkably larger than those of dataset2 (0.635 versus 0.615 and 0.646 versus 0.625). In fact, PRFLR(SNPs) outperformed any type of GRS for risk prediction in this dataset (all *P* ≤ 1.91E-03, Table 6).

Hosmer-lemeshow “goodness-of-fit” tests for model calibration are presented in Table 5. Models of wGRS2 showed borderline significance (*P* = 0.049), indicating that this model might not be well-calibrated. The remaining models were well-calibrated (*P* > 0.05). Calibration plots are provided in Supplementary Figure 3 for direct inspection. Therefore, wGRS2 was removed and wGRS1 was used in subsequent analysis in dataset2 for simplicity. We also explored the calibration of models in dataset3 (Table 5 and Supplementary Figure 4) and found all models to be well-calibrated (all *P* > 0.05) with the exception of wGRS2+fmc (*P* = 0.016) which was removed from further analyses in dataset3.

To further assess the potential value of genetic information in predicting glioma risk, we employed cNRI, which compares the shifts in reclassified categories by observed outcome and IDI analysis, which integrates net reclassification over all possible cut-offs for the probability of the outcome. As shown in Table 7, genetic information of any kind was superior to fmc and the improvement in percentage of correctly assigned subjects ranged from 28.2% for cGRS to 31.0% for PRFLR(SNPs) (all *P* < 0.0001). The differences in average predicted risks between cases and controls increased by 2.6% for cGRS, 2.7% for wGRS1 and 2.8% for PRFLR(SNPs) (all *P* < 0.0001). When added genetic information to baseline model, the updated models significantly improved cNRI (0.388 for cGRS+fmc, 0.342 for wGRS1+fmc and 0.336 for PRFLR(SNPs+fmc)) over fmc (all *P* < 0.0001). The IDI ranged from 3.4% for cGRS+fmc, 3.6% for wGRS1+fmc to 3.7% for PRFLR(SNPs+fmc) (all *P* < 0.0001). Llikewise, fmc also added reclassification benefit to genetic information: all cNRIs = 14.2% for cGRS, wGRS1 and PRFLR(SNPs), (all *P* = 0.0004). These results suggested fmc and genetic variants are independent risk factors for glioma and that genetic score does not capture the entirety of the information contained in fmc. wGRS1 also had significantly higher cNRI and IDI values over cGRS (*P* = 0.0385 for cNRI and *P* = 0.0380 for IDI) indicating that wGRS1 was superior to cGRS in the context of dataset2. These trends were validated in dataset3, where genetic information and fmc each added additional information for risk prediction (all *P* ≤ 0.0089), with the former having a significantly larger contribution to risk prediction than the latter (cNRI from 27.1% to 40.8% for genetic information over fmc all *P* < 0.0001; and cNRI = 9.8% for fmc over genetic information, all *P* = 0.0071). Incremental yield was also observed in IDI. Notably, the cNRI and IDI of PRFLR(SNPs) over fmc were higher compared with those of wGRS1 and cGRS (40.8% vs. 33.2% and 20.7% for cNRI; 5.8% vs. 3.4% and 3.4% for IDI). Adding PRFLR(SNPs) to baseline models yielded the great incremental value in cNRI (42%) and IDI (6.1%), suggesting that predicted risks from logistic regression analyses were more suitable for risk prediction in dataset3 than genetic risk score.

**Table 7:** Comparisons of cNRI and IDI in dataset2 and 3

Datasets	cGRS		wGRS1		PRFLR (SNPs)	
	cNRI 95%CI	IDI 95%CI	cNRI 95%CI	IDI 95%CI	cNRI 95%CI	IDI 95%CI
**Dataset2**						
fmc	0.282(0.180-0.385)	0.026(0.015-0.036)	0.291(0.189-0.394)	0.027(0.016-0.038)	0.310(0.208-0.413)	0.028(0.017-0.039)
cGRS			0.046(-0.058-0.150)	0.002(1e-04-0.003)	0.096(-0.008-0.200)	0.003(1e-04-0.006)
wGRS1					0.086(-0.011-0.183)	0.001(-6e-04-0.003)
PRFLR(SNPs)						
cGRS+fmc						
wGRS1+fmc						
**Dataset3**						
fmc	0.271(0.176-0.366)	0.034(0.025-0.044)	0.332(0.2371-0.427)	0.034(0.025-0.044)	0.408(0.314-0.502)	0.058(0.046-0.070)
cGRS			0.007(-0.089-0.102)	1e-04(-0.002-0.003)	0.196(0.102-0.290)	0.023(0.016-0.031)
wGRS1					0.197(0.103-0.290)	0.023(0.016-0.030)
PRFLR(SNPs)						
cGRS+fmc						
wGRS1+fmc						
	cGRS+fmc		wGRS1+fmc		PRFLR (SNPs+fmc)	
	cNRI 95%CI	IDI 95%CI	cNRI 95%CI	IDI 95%CI	cNRI 95%CI	IDI 95%CI
	0.388(0.287-0.488)	0.034(0.025-0.044)	0.342(0.239-0.444)	0.036(0.026-0.046)	0.336(0.234-0.438)	0.037(0.027-0.047)
	0.142(0.063-0.221)	0.009(0.004-0.014)	0.168(0.077-0.259)	0.010(0.005-0.016)	0.154(0.056-0.253)	0.012(0.006-0.017)
	0.077(-0.013-0.167)	0.007(0.002-0.012)	0.142(0.063-0.221)	0.009(0.004-0.014)	0.180(0.083-0.276)	0.010(0.005-0.015)
	-0.095(-0.198-0.008)	-0.006(-0.012--5e-4)	-0.151(-0.233--0.070)	-0.008(-0.013--0.002)	0.142(0.063-0.221)	0.009(0.004-0.014)
			0.040(-0.064-0.143)	0.002(-1e-04-0.003)	0.093(-0.011-0.197)	0.003(0-0.005)
					0.079(-0.016-0.175)	0.001(-6e-04-0.003)
	0.310(0.215-0.404)	0.038(0.029-0.047)	0.325(0.230-0.419)	0.038(0.029-0.047)	0.420(0.325-0.514)	0.061(0.050-0.073)
	0.098(0.027-0.168)	0.004(7e-04-0.007)	0.047(-0.048-0.141)	0.004(-2e-04-0.008)	0.236(0.143-0.330)	0.027(0.019-0.035)
	0.098(0.027-0.168)	0.004(6e-04-0.007)	0.098(0.027-0.168)	0.004(6e-04-0.007)	0.230(0.136-0.323)	0.027(0.019-0.035)
	0.161(0.066-0.257)	0.020(0.012-0.027)	0.130(0.034-0.226)	0.020(0.012-0.027)	0.098(0.027-0.168)	0.004(6e-04-0.006)
			0.004(-0.091-0.010)	0(-0.003-0.003)	0.197(0.103-0.290)	0.023(0.016-0.030)
					0.199(0.105-0.293)	0.023(0.016-0.030)

AUC: the area under operating characteristic curves; fmc: family history of caner; cGRS: count genetic risk score; wGRS: weighed genetic risk score; PRFLR: predicted risks from logistic regression analysis; cNRI: continuous net reclassification improvement; IDI: integrated discriminant index analysis. Statistics in each cell denotes comparison of variables in corresponding row over that of the corresponding column. Point estimation and 95%CIs were based on 2000 replicates of bootstrapping.

## DISCUSSION

In this three-stage designed study in a large Chinese population, five of the 15 SNPs identified in previous GWAS studies of European descent were reproducibly associated with glioma risk in dataset1 and 2. These SNPs were evaluated for predictive values by incorporating into a single statistic using different methods (i.e. cGRS, wGRS1, wGRS2 and PRFLR). We found genetic information to be an independent predictor of glioma risk and found it to add appreciable predictive value to baseline models toward classification of cases and controls. PRFLR captured most of the genetic information and outperformed GRSs in risk prediction, although the increase in AUC was modest compared with those of GRSs (AUC = 0.625 vs. 0.620 and 0.623). Dataset3 was used to both validate findings and establish models tailored for the Chinese population. Here, PRFLR+fmc served as the best model (AUC = 0.646). The potential benefit of adding genetic markers to risk models was further assessed by cNRI and IDI. Substantial and significant increases in cNRI and IDI were observed through the incorporation of genetic information (cNRI = 33.6% and 42.0%, IDI = 3.7% and 6.1% for PRFLR in dataset2 and dataset3, respectively). Of note, models were well-calibrated within these datasets.

Consistent with previously published reports, epidemiologic variables, such as cigarette smoking, were not associated with glioma risk in univariate logistic regression [23-25]. In fact, unlike many other types of cancer, epidemiologic and clinical parameters (i.e. cigarette smoking, alcohol consumption) have not been implicated as risk factors for glioma. During study recruitment, cases and controls were prospectively paired by age and sex. As a result, associations between age/sex and glioma risk were not observed in dataset2 and 3. Only family history was included in the baseline risk model. The interpretation of genetic variants is similar to that of family history. Genetic variants, however, contain more information and perform better (AUC = 0.615, 0.635 for PRFLR in dataset 2 and3, respectively). They are also independent risk factors for glioma, individually adding additional value to risk prediction (combined AUC = 0.625, 0.646 in dataset2 and3, respectively). These findings are in-line with those of Jostins and et al. [26], suggesting that both fmc and SNPs identified from GWAS captured only a subset of the genetic underpinnings of glioma. The reminder of the genetic determinants still remain unknown. A proportion of these may have been missed in our study due to our restrictive definition of a positive family history-having at least one first-degree relative with glioma. This metric provides only a crude estimate of familial risk and provides limited information about more nuanced family history.

Genetic variants as predictors have four advantages over clinical predictors: 1) they remain unchanged throughout one’s life, 2) they can be measured easily and accurately using a noninvasive saliva sample in a cost-effective manner, 3) they can be combined and used together in one prediction model, and 4) they can predict life-long risk while clinical factors only predict risk at a single time point [20, 26].

Three methods were used to generate a combined statistic for genetic variants in risk assessment. One, PRFLR, was simply based on predicted risks from logistic regression analysis. The other two, cGRS and wGRS were based on the concept of genetic risk score. We used two different methods of estimation as proposed by Meigs *et al* and Lin *et al* [11, 27]. We found cGRS and wGRS to have comparable discrimination ability in our study, a result that is discordant with findings from other studies [27-31]. We also found, PRFLR to outperform GRSs. These findings were further assisted by model assessment strategies (cNRI and IDI). Importantly, the results were validated by dataset3. GRSs are hypothesis based that effects are additive both within and between SNPs, while PRFLR is free from such assumption. As indicated by the definition, PRFLR uses predicted risks directly from logistic regression, while cGRS treats each SNP equally and uses count risk alleles of all SNPs and wGRS weighs each SNP by the genotypic OR and next, adds or multiplies the weighed genotypic risk together. cGRS is an extreme form of wGRS, both methods are hypothesis dependent and needs further manipulation of the data comparing with PRFLR, which might lead to loss of information and thus AUC losses.

To the best of our knowledge, this study is among the first to comprehensively explore the value of genetic information for risk prediction in glioma. It is worthy of mention that this is based on a three-stage design in a relatively large population. Such a design greatly reduces false positive findings and the possibility of overfitting and increases reliability. In addition, we didn’t carry the five SNPs straight into dataset3. In contrast, we interrogated the initial regions where association signals lie for comprehensive characterizing susceptible features of glioma and 7 independent SNPs were therefore harvested and used for risk prediction.

Overall, we began with a model based on consistently replicated SNPs identified from Caucasian GWAS and ended with a tailored model of SNPs identified to be associated with glioma risk well across the regions of initial signals in a Chinese population.

Concerns have been raised that substantial gains in risk prediction performance may not result in a substantial increase in AUC given that AUC does not contain information about the predicted risks [15, 32]. To address this issue, we also explored the added value of genetic variants to baseline model by NRI and IDI. These two measures offer incremental information over the AUC statistic. Because no established risk cut-offs exist for glioma at the moment, we chose continuous NRI for NRI estimation. The improvement in risk prediction afforded by genetic information was confirmed with more detailed characterization and comparison between performances of models incorporating genetic variants and family history. The matter of model calibration was addressed by H-L goodness-of-fit.

This study was based on findings from GWAS. Numerous studies have been carried out to explore susceptibility of glioma. These have largely featured candidate gene/pathway design and have implicated at least 61 SNPs thus far [33]. Attempts to validate these associations have yielded variable results and few genetic risk factors have been consistently replicated (except for those located in EGFR, CDKN2A and TP53). In contrast, SNPs identified from GWAS have been convincingly reproduced by multiple studies. We therefore began with SNPs identified from GWAS for this study, hoping to strength the validity of risk prediction.

There are several limitations in our study. First, one SNP in EGFR (rs2252586) which was associated with glioma in dataset1 was not available in dataset2. Therefore, its association could not be validated and this SNP was not included in prediction models in dataset2. Additional genotype data for EGFR was also unavailable in dataset3. Moreover, rs78378222 in the polyadenylation site of TP53 identified by fine-mapping studies in Caucasian populations to be associated glioma risk [34, 35]. Another common SNP rs1920116 located in intron region of *TERC*, were first identified to be associated glioma risk by Walsh et al in 2014 in Caucasian population [36] and then replicated in a Chinese population by Wang et al in 2015 [37]. However, these findings published after the implementation of our study, therefore rs78378222 and rs1920116 were not included in our study either. This omission may affect the estimates of AUC statistics, cNRI and IDI of the final models. Second, GWAS that underpin our study were all based on Caucasian populations. It is possible that other SNPs in these regions or novel regions may be important in Chinese population. We overcome the former issue in dataset3 of our study, which evaluates SNPs perfectly cover the regions where association signals lie. For the latter, no GWAS in Chinese populations has been reported till now and the issue was not able to address in this study. Third, AUC in the best scenario in our study was 0.646, far from potential consideration of clinical utility (AUC ≥ 0.8). Indeed, the underlying architecture of genetic susceptibility to glioma may not include as large a proportion of common variants as has been seen for other cancers to date. Furthermore, it is possible that the addition of yet unidentified rare risk alleles with large effects could improve discrimination. One such SNP, rs55705857 at 8q24.21 was identified through imputation effort combined with next generation sequencing in Caucasian population [38, 39]. After all, risk model prediction is not a diagnostic tool but rather provides an estimate of the likelihood of developing disease in the future. Fourth, exposure to therapeutic doses or high-dose radiation is the most firmly established environmental risk factor for the development of glioma [40]. In our three datasets, only 41 cases and 17 controls reported professional exposure to ionizing radiation, far from sufficient for statistical analysis. Our study was therefore ill-equipped to quantify the role of ionizing radiation in risk prediction of glioma. This, unfortunately, may substantially diminish the clinical relevance of the model we report. Last but not least, glioma is heterogeneous, encompassing a wide spectrum of subtypes (astrocytoma, oligodendroglioma, mixed oliogoastrocytoma, and ependymoma) [41]. Studies have suggested different subgroups of glioma may represent distinct pathological entities. For example, genetic variants specific to each of the subtypes have also been found in multiple studies [25, 42-44]. Therefore, it is better to build risk prediction models respectively for each subtype based on their unique susceptible features. Finally, the conclusions of this study may be influenced by the prevalence of the disease under study, as AUC statistic does not incorporate disease incidence as a parameter.

## SUBJECTS AND METHODS

### Study population

This study consists of three case-control datasets. Flow diagrams of the enrollment of the study populations are presented in Supplementary Figure 1. Detailed flowchart of the study design is presented in Supplementary Figure 2. Demographic characteristics and clinical features of each study population are presented Supplementary Table 1. Overall, all subjects were genetically unrelated ethnic Han Chinese. Case subjects were newly diagnosed and pathologically confirmed gliomas patients from the Department of Neurosurgery at Huashan Hospital and Changzheng Hospital (Shanghai, China). These patients were recruited consecutively into each of the study dataset without restrictions on age, gender and histology. Those who had self-reported cancer history other than glioma, previous cancers, metastasized cancer from other organs, spinal gliomas, and previous radiotherapy or chemotherapy were excluded. In total, 992, 976 and 983 eligible cases each provided an informed consent and were enrolled for dataset1 [45, 46], 2 [8] and 3 [47-49], respectively.

Cancer-free controls were selected from visitors undergoing routine physical examination and trauma patients at the emergency medical center. For dataset2 and 3, 1057 and 1024 controls were enrolled at the same hospitals during the same time periods as case enrollment. Dataset1 consisted of two parts: 1008 from four districts and counties of Shanghai, described elsewhere [50], and 1245 from communities of Nanjing surrounding areas which were primary used for lung cancer GWAS [51]. All controls had no known central nervous system-related diseases, self-reported history of any cancer or history of radiotherapy/chemotherapy at the time of recruitment.

Controls of dataset2 and 3 were frequency matched to case subjects according to age (within 5 years), sex and geographic origins, while controls differed from cases by demographic data (mainly gender and age) in dataset1. Therefore, due to 1) the relatively poor quality of the epidemiological information in controls, 2) relatively larger sample size, 3) consistent frequencies of selected SNPs between Nanjing and Shanghai subsets (data not shown), dataset1 was only used for identifying significant associations between SNPs and glioma risk and serves as a spur to introduce dataset2 and 3 for model development.

Each subject was interviewed face-to-face by trained personnel using a questionnaire, which we have described previously [52]. After the interview, each subject provided 3–5 mL venous blood. This study were approved by School of Life Sciences of Fudan University Ethics Board. All experiments were carried out in accordance with approved guidelines of School of Life Sciences of Fudan University (Shanghai, China).

### SNP selection, genotyping and quality controls

15 previously identified SNPs^3-5,7^, representing 6 distinct loci, were selected for dataset1 and 2. In dataset3, for the consistently replicated SNPs in both dataset1 and 2, we extended the chromosome regions where these signals lie and selected tag SNPs in Chinese population according to HapMap database (http://www.hapmap.org/, phase III Aug 10, on NCBI B36 assembly, dbSNP b132; population: Han Chinese in Beijing, China). That is, for SNP selection in dataset3, those 6 targeted chromosome region at least covered the complete LD where those consistently replicated SNPs lie. Haploview program 4.2 was used for the selection on basis of pairwise LD r^2^ threshold of 0.8 and minor-allele frequency (MAF) ≥0.05. Within these well encompassed regions, 42 SNPs were chosen for dataset3, including 14 at 5p15.33, 15 at 11q23.3 and 13 at 20q13.33. Genotyping was performed by MassARRAY iPLEX system (Sequenom, Inc.).

Samples were removed if their genotype rate was <95%. SNPs were excluded if they had: (i) call rate <95%; (ii) MAF <0.05; or (iii) *P*<0.01 for Hardy-Weinberg Equilibrium test among controls. Moreover, individuals were removed from multivariate logistic analysis and model development if they missed any one of the genotypes.

### Statistical analyses

#### Selection of clinical variables and SNPs for model development

Associations between SNPs, clinical variables and risk of glioma were estimated by computing ORs and their 95% CIs using a univariate logistic regression model. Log-additive model was used to derive genotype relative risks from the allelic OR. The first series of models were built on dataset2. Only SNPs that showed consistently significant association with glioma risk in both dataset1 and 2 were qualified for model development. The second series of models were constructed on dataset3. To ensure the independent effect of each SNPs, we only selected SNPs if they had (i) *P*<0.05 for association with glioma risk; (ii) pairwise r^2^<0.35; (iii) remained in a multivariate logistic regression model using backward likelihood ratio method. Fmc was also included in model development for its independency association with glioma risk.

#### Risk model development

There were three approaches for incorporating SNPs into a risk prediction model. The first approach simply used predicted risks from logistic regression analysis (PRFLR). The other two were based on genetic risk score (GRS): a simple risk allele count method (count GRS, cGRS) where the number of risk alleles were summed for each individual and a weighted method based on the effect sizes (genotypic OR) (weighted GRS, wGRS) [11, 27, 28, 30]. For wGRS, there were two different methods to model. One (wGRS1) was generated according to following equation: wGRS1 = w_1_×SNP_1_+w_2_×SNP_2_+. . .+ w_k_×SNP_k_, where SNP_i_ denoted the number of risk alleles for the specific SNPs(SNP_i_=0,1,2), w_i_ was the appropriate weight of each SNP, in our study, w_i_ equaled to the allelic OR_i_, and k was the number of SNPs used [27]. The other (wGRS2) was generated by multiplying risks of the genotypic OR of each individual SNP. Briefly, for each of the three genotypes at each SNP, we converted the genotype relative risk to the risk relative to the average risk of population. Then the overall risk relative to the population was derived by multiplying the risks relative to the population of all SNPs [28, 29]. The formula was: wGRS2= SNP_1_ × SNP_2_ ×. . .× SNP_k_, where SNP_k_ was overall risk for the k_th_ SNP. All three GRS approaches were based on the assumptions that no interaction existed among SNPs and that they each had independent effects [53]. We tested interactions for each pair of SNPs by including both main effects and an interaction term (a product of two main effects) in a logistic regression analysis [54]. Finally, PRFLR, cGRS and wGRS were used to construct receiver-operating characteristic (ROC) and AUC in dataset2 and 3. To ensure reliability of data, we excluded subjects from model development if they had missing information on any of the predictors.

#### Assessment of the performances of risk models

The differences in AUC between two models was tested by DeLong’s test [55]. The H-L test was used as a calibration statistic to examine the goodness of fit of the models [56]. Calibration quantifies how closely the predicted probabilities of an event match the actual experience. Two thousand replicates of bootstrap were carried out as internal validation of models to adjust for potential overfitting. Furthermore, continuous NRI (cNRI) and IDI were used to calculate the incremental value added of genetic information to the prediction of glioma risk [57, 58]. cNRI does not require any discrete risk categories and relies on the proportions of cases correctly assigned a higher probability and controls correctly assigned a lower probability by an updated model compared with the initial model. IDI equaling x% means that the difference in average predicted risks between cases and controls increased by x% in the updated model. cNRI and IDI were estimated and tested for significance using methods proposed by Pencina *et al* [57]. All *P* values were two-sided, and *P* values < 0.05 were considered statistically significant. All statistical analyses were done in R version 3.0.1 (R Foundation for Statistical Computing, Vienna, Austria) using ROCR, rms, Hmisc, epitools and PredictABEL packages [59, 60].

## SUPPLEMENTARY MATERIALS FIGURES AND TABLES




