# Supplementary material for: Development of risk prediction models for glioma based on genome-wide association study findings and comprehensive evaluation of predictive performances
Source: Oncotarget. 2016 Jul 28;9(9):8311–25. doi: 10.18632/oncotarget.10882 (PMC5823595; doi:10.18632/oncotarget.10882)
Supplement: Supplementary file 1 [file oncotarget-09-8311-s001.pdf]

Development of risk prediction models for glioma based on genome-wide association study findings and comprehensive evaluation of predictive performances

SUPPLEMENTARY DATA

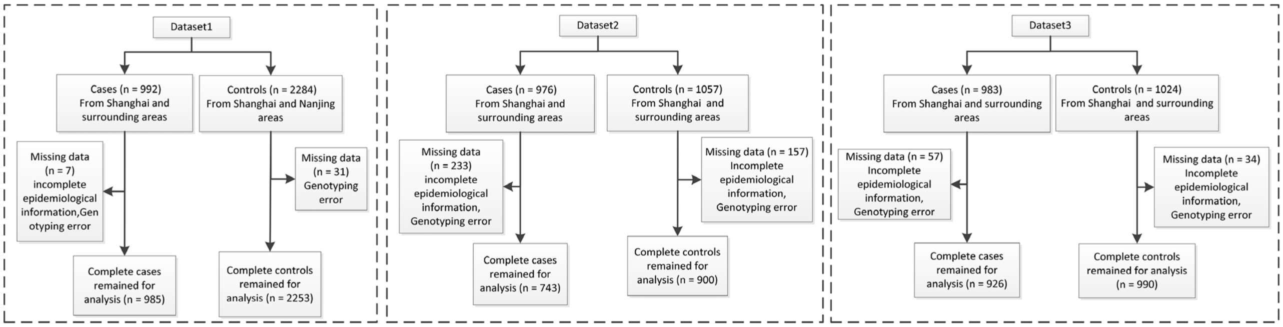

Supplementary Figure 1: Participant flow diagrams of the three datasets in this study.

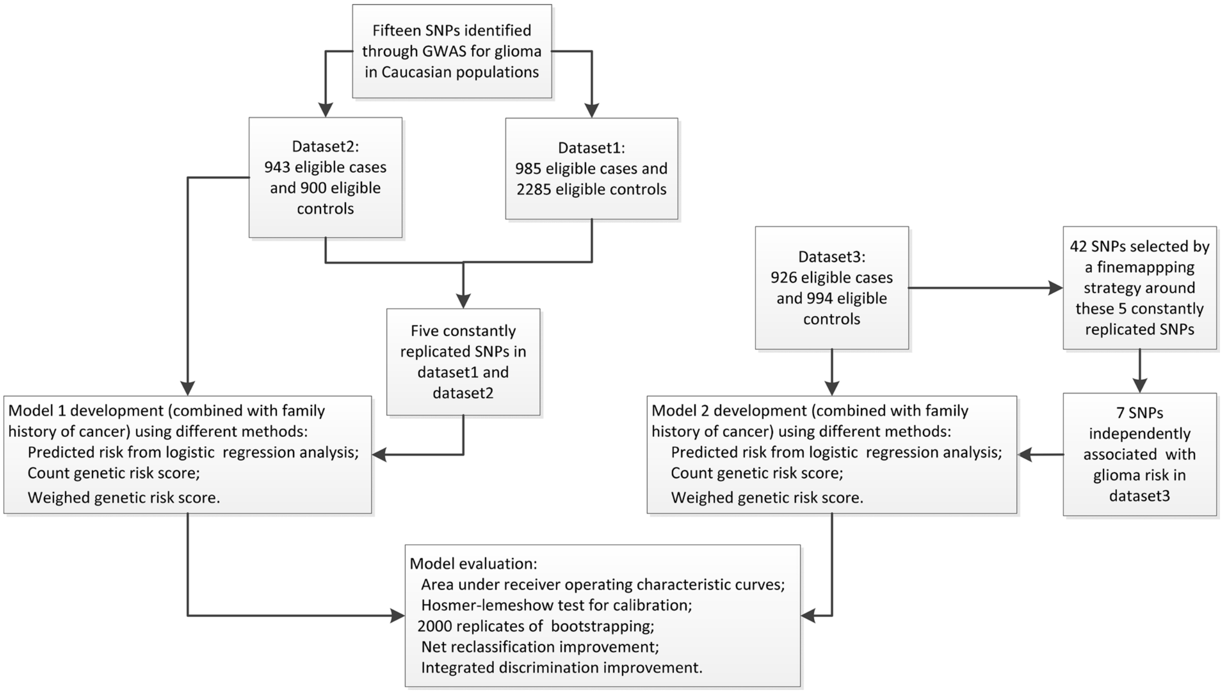

Supplementary Figure 2: Flow chart of the study design.

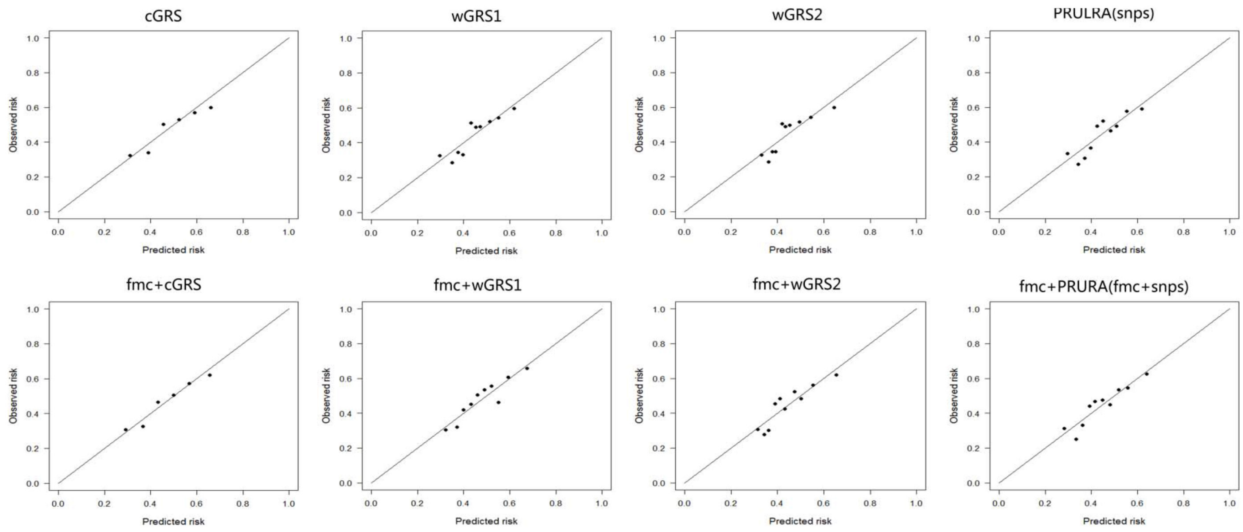

Supplementary Figure 3: Calibration plots in dataset2.
fmc: family history of caner; cGRS: count genetic risk score; wGRS: weighed genetic risk score; PRFLR: predicted risks from logistic regression analysis.

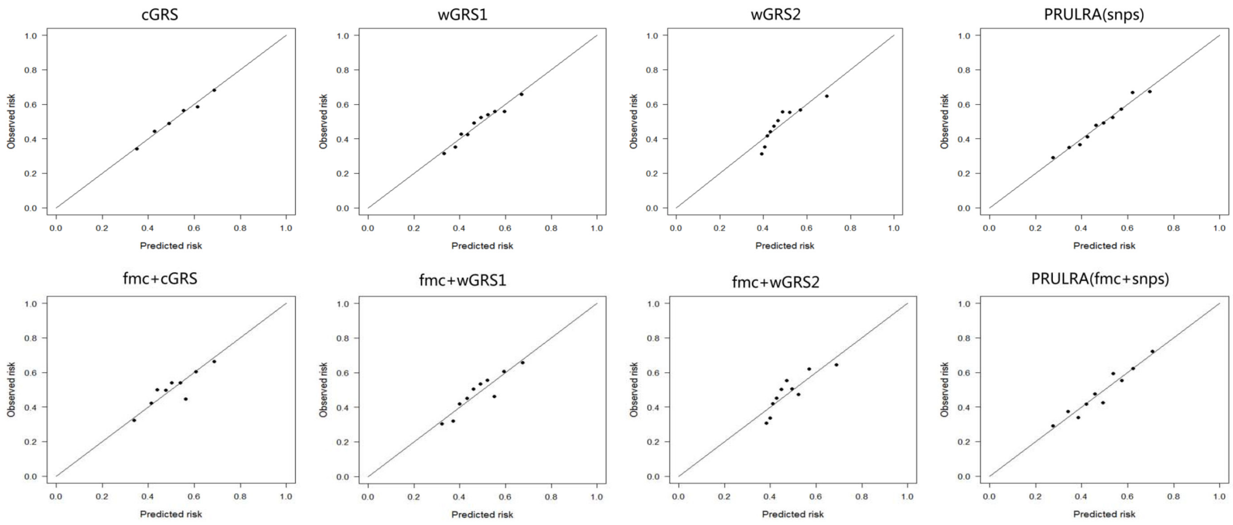

Supplementary Figure 4: Calibration plots in dataset3.
fmc: family history of caner; cGRS: count genetic risk score; wGRS: weighed genetic risk score; PRFLR: predicted risks from logistic regression analysis.

Supplementary Table 1: Basic epidemiological information of the study subjects

See Supplementary File : 1

Supplementary Table 2: Univariate logistic regression analysis of family history of caner with glioma risk

Epidemiological variable	Dataset2		Dataset3	
	OR (95%CI)	*P*	OR (95%CI)	*P*
Family history of cancer	1.63(1.24-2.15)	0.001	1.47(1.13-1.91)	0.004

Supplementary Table 3: Associations between SNPs selected from previous GWAS and gioma risk in datasets 1 and

See Supplementary File: 1

Supplementary Table 4: Associations between selected SNPs and glioma risk in dataset3

See Supplementary File: 1

Supplementary Table 5: Interaction test P values for all combinations of 5 SNPs used for model development in dataset2

SNP1	SNP2	Interaction P
rs2736100	rs2736100	0.326
rs2736100	rs2157719	0.536
rs2736100	rs498872	0.782
rs2736100	rs6010620	0.546
rs1077236	rs2157719	0.769
rs1077236	rs498872	0.831
rs1077236	rs6010620	0.237
rs2157719	rs498872	0.386
rs2157719	rs6010620	0.499
rs498872	rs6010620	0.228

Supplementary Table 6: Interaction test P values for all combinations of 7 SNPs used for model development in dataset3

SNP1	SNP2	Interaction P
rs2853677	rs2735948	0.531
rs2853677	rs6589664	0.427
rs2853677	rs494560	0.785
rs2853677	rs17748	0.342
rs2853677	rs3761121	0.196
rs2853677	rs1058319	0.261
rs2735948	rs6589664	0.125
rs2735948	rs494560	0.715
rs2735948	rs17748	0.700
rs2735948	rs3761121	0.031
rs2735948	rs1058319	0.199
rs6589664	rs494560	0.746
rs6589664	rs17748	0.333
rs6589664	rs3761121	0.436
rs6589664	rs1058319	0.055
rs494560	rs17748	0.351
rs494560	rs3761121	0.311
rs494560	rs1058319	0.380
rs17748	rs3761121	0.963
rs17748	rs1058319	0.949
rs3761121	rs1058319	0.059
