# Supplementary material for: Development of risk prediction models for glioma based on genome-wide association study findings and comprehensive evaluation of predictive performances
Source: Oncotarget. 2016 Jul 28;9(9):8311–25. doi: 10.18632/oncotarget.10882 (PMC5823595; doi:10.18632/oncotarget.10882)
Supplement: Supplementary file 2 [file oncotarget-09-8311-s002.doc]

Supplementary Table 1. Basic epidemiological information of the study subjects

|  |  | Dataset1 | | |  | Dataset2 | |  |  | Dataset3 | |  |
| --- | --- | --- | --- | --- | --- | --- | --- | --- | --- | --- | --- | --- |
| Variables | | Cases  (985) | Controls  (1008)^a^ | Controls  (1246)^b^ |  | Cases  (976) | Controls  (1057) | P Value from x^2^ Test |  | Cases  (983) | Controls  (1024) | P Value from x^2^ Test |
|  |  | NO.% | NO.% | NO.% |  | NO.% | NO.% |  |  | NO.% | NO.% |  |
| Sex | |  |  |  |  |  |  | 0.617 |  |  |  | 0.550 |
| Male | | 575(58.4) | 1008(100) | 882(66.0) |  | 581(59.5) | 633(59.9) |  |  | 579(58.8) | 623(60.9) |  |
| Female | | 391(40.7) | 0 (0) | 424(34.0) |  | 280(37.7) | 367(40.8) |  |  | 390(39.6) | 397(38.7) |  |
| Missing data | |  |  |  |  | 18(1.7) | 5(0.5) |  |  | 14(1.5) | 4(3.9) |  |
| Mean age, years(SD) | | 42.8±13.5 | 62.1±10.0 | 41.8±16.3 |  | 42.3±16.3 | 42.1±18.3 |  |  | 42.2±18.6 | 42.1±15.8 |  |
| Age group | |  |  |  |  |  |  | 0.126 |  |  |  | 0.092 |
| Children(≤18 years) | | 69(7.0) | 0(0) | 98(7.9) |  | 80(8.2) | 68(6.4) |  |  | 86(8.8) | 91(8.9) |  |
| Adults(>18 years) | | 916(93.0) | 1008(100) | 1148(92.1) |  | 896(91.8) | 989(93.6) |  |  | 887(91.2) | 928(90.6) |  |
| Cigarette smoking | |  | NA | NA |  |  |  | 0.229 |  |  |  | 0.911 |
| Non-smokers | | 496(50.4) |  |  |  | 605(62.0) | 625(59.1) |  |  | 587(59.7) | 602(58.8) |  |
| Smokers | | 416(42.2) |  |  |  | 370(38.0) | 413(39.1) |  |  | 387(39.4) | 412(40.2) |  |
| Missing data | | 73(7.4) |  |  |  | 11(1.1) | 19(1.8) |  |  | 9(0.9) | 10(1.0) |  |
| Family history of cancer  first-degree relatives | |  | NA | NA |  |  |  | 0.003 |  |  |  | 0.004 |
| No | | 675(68.5) |  |  |  | 682(70.2) | 803(76.0) |  |  | 714(72.6) | 793(77.4) |  |
| Yes | | 190(19.3) |  |  |  | 170(17.5) | 138(13.1) |  |  | 190(19.3) | 122(11.9) |  |
| Missing | | 120(12.2) |  |  |  | 124(12.4) | 116(11.0) |  |  | 109(11.0) | 109(10.6) |  |
| Histologic type | |  |  |  |  |  |  |  |  |  |  |  |
| Glioblastoma | | 315(32.0) |  |  |  | 312(32.2) |  |  |  | 303(30.8) |  |  |
| Astrocytomas except  for glioblastoma^c^ | | 415(42.1) |  |  |  | 360(37.2) |  |  |  | 306(31.1) |  |  |
| Other glioma^d^ | | 243(24.7) |  |  |  | 296(30.6) |  |  |  | 369(37.5) |  |  |
| Missing data | | 12(1.2) |  |  |  | 8(0.8) |  |  |  | 5(0.5) |  |  |

a, controls from Shanghai areas; b, controls from Nanjing areas; NA: not available; c, Astrocytomas except for glioblastoma including diffuse astrocytomas, anaplastic astrocytomas and other astrocytomas; d, Other gliomas including oligodendrogliomas, enpendymomas or mixed gliomas.

| Supplementary Table 3. Associations between SNPs selected from previous GWAS and gioma risk in datasets 1 and 2 | | | | | | | | | | | | |
| --- | --- | --- | --- | --- | --- | --- | --- | --- | --- | --- | --- | --- |
| SNP | CHR. | Nearest gene | Region | Location on | Non-risk | Risk | Dataset1 | | | Dataset2 | | |
|  |  |  |  | Chromosome^a^ |  |  | Risk allele frequency | OR(95%CI)^b^ | *P* value^b^ | Risk allele frequency | OR (95%CI)^b^ | *P* value^b^ |
|  |  |  |  |  |  |  | Cases Controls |  |  | Cases Controls |  |  |
| rs2736100 | 5 | *TERT* | Intron | 1339516 | T | G | 0.479 0.413 | 1.30(1.17-1.46) | 3.96E-06 | 0.482 0.418 | 1.29(1.13-1.49) | 2.69E-04 |
| rs2736098 | 5 | *TERT* | Exon | 1347086 | G | A | 0.393 0.375 | 1.08(0.93-1.24) | 0.306 | 0.404 0.346 | 1.28(1.11-1.48) | 5.89E-04 |
| rs10464870 | 8 | *CCDC26* | Intergenic | 130547005 | T | C | 0.178 0.177 | 1.00(0.84-1.20) | 0.987 | 0.172 0.173 | 0.99(0.83-1.19) | 0.896 |
| rs891835 | 8 | *CCDC26* | Intergenic | 130560934 | G | T | 0.862 0.872 | 1.08(0.75-1.13) | 0.422 | 0.886 0.872 | 1.15(0.71-1.08) | 0.203 |
| rs1077236 | 8 | *CCDC26* | Intergenic | 130709683 | A | C | 0.698 0.677 | 1.10(0.95-1.28) | 0.219 | 0.725 0.688 | 1.20(1.03-1.39) | 0.021 |
| rs4295627 | 8 | *CCDC26* | Intergenic | 130754639 | G | T | 0.740 0.724 | 1.08(0.95-1.23) | 0.214 | 0.762 0.739 | 1.13(0.75-1.03) | 0.121 |
| rs1063192 | 9 | *CCDKN2A/B* | Exon | 21993367 | T | C | 0.216 0.188 | 1.19(1.00-1.41) | 0.049 | 0.195 0.180 | 1.11(0.93-1.32) | 0.267 |
| rs2157719 | 9 | *CCDKN2A/B* | Intron | 22023366 | T | C | 0.140 0.113 | 1.28(1.08-1.51) | 4.19E-03 | 0.141 0.111 | 1.32(1.07-1.62) | 9.23E-03 |
| rs1412829 | 9 | *CCDKN2A/B* | Intron | 22033926 | A | G | 0.140 0.113 | 1.28(1.08-1.51) | 4.04E-03 | 0.140 0.114 | 1.27(1.03-1.56) | 0.025 |
| rs4977756 | 9 | *CCDKN2A/B* | Intron | 22058652 | C | T | 0.228 0.214 | 1.09.(0.95-1.25) | 0.200 | 0.223 0.210 | 1.08(0.92-1.28) | 0.352 |
| rs498872 | 11 | *PHLDB1* | UTR-5 | 117982577 | A | G | 0.303 0.272 | 1.23(1.08-1.39) | 1.19E-03 | 0.349 0.285 | 1.35(1.16-1.56) | 7.81E-05 |
| rs6010620 | 20 | *RTEL1* | Intron | 61780283 | T | C | 0.304 0.266 | 1.21(1.07-1.37) | 2.39E-03 | 0.330 0.267 | 1.35(1.16-1.57) | 9.25E-05 |
| rs4809324 | 20 | *RTEL1* | Intron | 61788664 | A | G | 0.132 0.107 | 1.27(1.02-1.57) | 0.030 | 0.144 0.121 | 1.23(1.00-1.50) | 0.047 |
| rs2252586 | 7 | *EGFR* | Intergenic | 54946418 | C | T | 0.026 0.027 | 0.99(0.63-1.53) | 0.947 | NA | NA | NA |
| rs1005176 | 7 | *EGFR* | Intergenic | 55004391 | G | C | 0.324 0.273 | 1.28(1.10-1.49) | 0.002 | NA | NA | NA |

a, based on NCBI Build 36; b, Odds ratios (ORs), 95% confidence interval (95%CI) and P values were calculated from univariate logistic regression analyses based on additive model; NA: not available In dataset1, associations of 7 SNPs (rs2736100, rs4295627, rs2157719, rs1412829, rs4977756, rs498872, rs6010620) were calculated based on controls from both Nanjing and Shanghai areas; those of the other 8 were calculated based solely on controls of Shanghai area duo to availability of data, while cases remains consistent.

| Supplementary Table 4. Associations between selected SNPs and glioma risk in dataset3 | | | | | | | | | |
| --- | --- | --- | --- | --- | --- | --- | --- | --- | --- |
| SNP | CHR | Nearest gene | Region | Location on | Non-risk | Risk | Risk allele frequency | OR (95%CI)^b^ | *P* value^b^ |
|  |  |  |  | Chromosome ^a^ |  |  | Cases Controls |  |  |
| rs7445640 | 5 | *SLC6A18* | Intron | 1236212 | T | G | 0.231 0.245 | 0.93(0.80-1.07) | 0.308 |
| rs13361701 | 5 | *SLC6A18* | Intron | 1243427 | T | G | 0.010 0.004 | 2.42(1.05-5.58) | 0.032 |
| rs4073918 | 5 | *SLC6A18* | Exon | 1244425 | G | A | 0.282 0.290 | 0.96(0.84-1.11) | 0.600 |
| rs2736118 | 5 | *TERT* | Intron | 1260195 | A | G | 0.063 0.055 | 1.17(0.89-1.52) | 0.265 |
| rs4246742 | 5 | *TERT* | Intron | 1267356 | A | T | 0.384 0.378 | 1.03(0.90-1.17) | 0.673 |
| rs4975605 | 5 | *TERT* | Intron | 1275528 | C | A | 0.081 0.087 | 0.93(0.74-1.17) | 0.532 |
| rs2853677 | 5 | *TERT* | Intron | 1287194 | T | C | 0.449 0.375 | 1.36(1.20-1.55) | 2.70E-06 |
| rs2735948 | 5 | *TERT* | Intergenic | 1299213 | C | T | 0.170 0.146 | 1.20(1.01-1.42) | 0.044 |
| rs2853668 | 5 | *TERT* | Intergenic | 1300025 | C | A | 0.308 0.292 | 1.08(0.94-1.24) | 0.281 |
| rs4975612 | 5 | *TERT* | Intergenic | 1300310 | G | T | 0.491 0.496 | 0.98(0.87-1.11) | 0.778 |
| rs4635969 | 5 | *CLPTM1L* | Intergenic | 1308552 | C | T | 0.101 0.010 | 1.03(0.83-1.27) | 0.813 |
| rs6554759 | 5 | *CLPTM1L* | Intergenic | 1317102 | A | G | 0.058 0.046 | 1.27(0.95-1.69) | 0.100 |
| rs401681 | 5 | *CLPTM1L* | Intron | 1322087 | C | T | 0.312 0.305 | 1.03(0.90-1.19) | 0.640 |
| rs414965 | 5 | *CLPTM1L* | Intron | 1324121 | C | T | 0.169 0.147 | 1.18(0.99-1.40) | 0.061 |
| rs6589664 | 11 | *TMEM25* | Exon | 117910014 | G | A | 0.310 0.271 | 1.21(1.05-1.39) | 6.80E-03 |
| rs12289253 | 11 | *TMEM25* | Exon | 117910278 | G | A | 0.509 0.477 | 1.13(1.00-1.29) | 0.052 |
| rs3741324 | 11 | *TMEM25* | *UTR-3* | 117911045 | A | G | 0.565 0.523 | 0.84(1.04-1.34) | 9.18E-03 |
| rs10736492 | 11 | *ARCN1* | *Intron* | 117964479 | A | G | 0.258 0.240 | 1.10(0.94-1.27) | 0.206 |
| rs7115634 | 11 | *ARCN1* | *Intron* | 117971459 | G | A | 0.565 0.508 | 0.79(1.11-1.43) | 3.68E-04 |
| rs604096 | 11 | *ARCN1* | Intergenic | 117980308 | C | T | 0.738 0.710 | 0.88(1.00-1.32) | 0.056 |
| rs2236661 | 11 | *PHLDB1* | *Intron* | 118004604 | G | C | 0.266 0.209 | 1.37(1.18-1.60) | 3.43E-05 |
| rs12419235 | 11 | *PHLDB1* | *Intron* | 118011767 | G | T | 0.171 0.164 | 1.05(0.88-1.24) | 0.601 |
| rs494560 | 11 | *PHLDB1* | *Intron* | 118026759 | A | G | 0.801 0.746 | 0.73(1.18-1.60) | 4.04E-05 |
| rs17748 | 11 | *PHLDB1* | *UTR-3* | 118033634 | C | T | 0.327 0.263 | 1.36(1.18-1.56) | 1.57E-05 |
| rs2276064 | 11 | *TREH* | Exon | 118034913 | G | A | 0.409 0.418 | 0.96(0.85-1.10) | 0.581 |
| rs10892251 | 11 | *TREH* | *Intron* | 118048773 | C | T | 0.325 0.279 | 1.24(1.08-1.43) | 2.15E-03 |
| rs11216943 | 11 | *LOC768086* | Intergenic | 118061608 | G | A | 0.284 0.239 | 1.26(1.09-1.45) | 1.73E-03 |
| rs4639966 | 11 | *LOC768086* | Intergenic | 118078729 | C | T | 0.724 0.682 | 0.82(1.07-1.41) | 3.98E-03 |
| rs496547 | 11 | *LOC768086* | Intergenic | 118081673 | T | A | 0.253 0.244 | 1.05(0.91-1.22) | 0.506 |
| rs6089953 | 20 | *RTEL1* | Intron | 62291008 | A | G | 0.320 0.265 | 1.31(1.14-1.51) | 1.47E-04 |
| rs2738780 | 20 | *RTEL1* | Intron | 62299814 | C | T | 0.111 0.117 | 0.94(0.77-1.15) | 0.573 |
| rs6062484 | 20 | *RTEL1* | Intron | 62301980 | T | C | 0.191 0.141 | 1.43(1.21-1.70) | 3.55E-05 |
| rs2297437 | 20 | *RTEL1* | Intron | 62305274 | G | A | 0.155 0.141 | 1.11(0.93-1.33) | 0.241 |
| rs3787098 | 20 | *RTEL1* | Intron | 62307761 | G | A | 0.163 0.127 | 1.34(1.12-1.61) | 1.32E-03 |
| rs6089956 | 20 | *RTEL1* | Intron | 62308364 | C | A | 0.303 0.317 | 0.94(0.82-1.08) | 0.367 |
| rs2297440 | 20 | *RTEL1* | Intron | 62312299 | T | C | 0.323 0.258 | 1.37(1.19-1.58) | 8.36E-06 |
| rs2738788 | 20 | *ARFRP1* | Intron | 62332436 | A | G | 0.765 0.741 | 0.88(0.98-1.32) | 0.087 |
| rs2257885 | 20 | *ARFRP1* | Intron | 62334220 | A | G | 0.360 0.274 | 1.50(1.31-1.72) | 7.34E-09 |
| rs3761121 | 20 | *ZGPAT* | Intron | 62342695 | A | G | 0.269 0.202 | 1.45(1.25-1.69) | 9.85E-07 |
| rs1058319 | 20 | *SLC2A4RG* | UTR-3 | 62374389 | T | C | 0.354 0.256 | 1.59(1.39-1.83) | 4.76E-11 |
| rs5019252 | 20 | *ZBTB46* | Exon | 62378349 | C | T | 0.458 0.368 | 1.45(1.27-1.65) | 1.89E-08 |
| rs4809224 | 20 | *ZBTB46* | Intron | 62387830 | T | A | 0.128 0.135 | 0.94(0.78-1.13) | 0.499 |

a, based on NCBI Build 36; b, ORs, 95%CI and *P* values were calculated from univariate logistic regression analysis based on additive model.
